# Removing duplicate reads using graphics processing units

**DOI:** 10.1186/s12859-016-1192-5

**Published:** 2016-11-08

**Authors:** Andrea Manconi, Marco Moscatelli, Giuliano Armano, Matteo Gnocchi, Alessandro Orro, Luciano Milanesi

**Affiliations:** 1 Institute for Biomedical Technologies, National Research Council, Via Fratelli Cervi, 93, Segrate (Mi), 20090 Italy; 2 Department of Electrical and Electronic Engineering, University of Cagliari, P.zza D’Armi, Cagliari (CA), 09123 Italy

**Keywords:** Next generation sequencing, Duplicate reads, Graphics processing units, CUDA

## Abstract

**Background:**

During library construction polymerase chain reaction is used to enrich the DNA before sequencing. Typically, this process generates duplicate read sequences. Removal of these artifacts is mandatory, as they can affect the correct interpretation of data in several analyses. Ideally, duplicate reads should be characterized by identical nucleotide sequences. However, due to sequencing errors, duplicates may also be nearly-identical. Removing nearly-identical duplicates can result in a notable computational effort. To deal with this challenge, we recently proposed a GPU method aimed at removing identical and nearly-identical duplicates generated with an Illumina platform.

The method implements an approach based on prefix-suffix comparison. Read sequences with identical prefix are considered potential duplicates. Then, their suffixes are compared to identify and remove those that are actually duplicated.

Although the method can be efficiently used to remove duplicates, there are some limitations that need to be overcome. In particular, it cannot to detect potential duplicates in the event that prefixes are longer than 27 bases, and it does not provide support for paired-end read libraries. Moreover, large clusters of potential duplicates are split into smaller with the aim to guarantees a reasonable computing time. This heuristic may affect the accuracy of the analysis.

**Results:**

In this work we propose GPU-DupRemoval, a new implementation of our method able to (*i*) cluster reads without constraints on the maximum length of the prefixes, (*ii*) support both single- and paired-end read libraries, and (*iii*) analyze large clusters of potential duplicates.

**Conclusions:**

Due to the massive parallelization obtained by exploiting graphics cards, GPU-DupRemoval removes duplicate reads faster than other cutting-edge solutions, while outperforming most of them in terms of amount of duplicates reads.

## Background

Duplicate reads are one of the most problematic artifacts generated during polymerase chain reaction amplification. Ideally, duplicates should have identical nucleotide sequences. However, due to sequencing errors, they may end up to be nearly-identical [1]. Duplicates can affect the accuracy of some analyses on NGS data. Removal of these artifacts can be an essential pre-processing step, in particular on applications based on resequencing. For instance, in SNP calling, errors introduced in early amplification steps are shared by PCR duplicates, making very difficult to distinguish between repeated (but identical) errors and real SNPs [2, 3]. Duplicate removal is also a mandatory step to detect CNVs using read-depth (RD) based methods [4]. These methods assume that the RD in a genomic region depends on the copy number of that region. As a consequence, duplicates need to be detected and removed to avoid incorrect read count. Duplicates can also affect the accuracy of de-novo sequencing. During scaffolding, paired-end reads are mapped on contigs with the aim to rank their order. In this phase, two contigs are considered connected depending on the number of read pairs that link them (the higher the number the stronger the connection). Hence, PCR duplicates may result in false-positive connections between contigs.

Duplicate sequences can be natural or artificial. Ideally, only artificial duplicates should be removed, while natural ones should be retained. Unfortunately, natural and artificial duplicate sequences are indistinguishable. This is the reason why a fraction of reads labeled as duplicates may in fact be generated from distinct molecules, yielding a loss of natural reads. However, this situation occurs typically during the analysis of single-end reads. In fact, as for paired-end reads, the probability of finding independent molecules identical at both ends being very low [5].

Removal tools proposed in the literature implement methods that focus either on alignment-based or on alignment-free strategies. Alignment-based tools assume that duplicate reads will be mapped to the same position on a reference genome. These tools analyze the alignments obtained by running an embedded procedure (or a third-party aligner) with the goal of finding reads with identical mapping coordinates. These reads are analyzed and those that meet predefined quality constraints are classified as duplicates. The performance of these tools is affected by the alignment constraints and by the accuracy of the aligner. Moreover, it should be pointed out that these tools cannot be used in absence of a complete reference genome.

Picard MarkDuplicates [6], samtools rmdup [7], and SEAL [8] are tools that implement an alignment-based strategy. Picard MarkDuplicates identifies duplicates by analyzing the alignments generated by a third-party aligner. As for paired-end reads, it finds the 5’ coordinates and mapping orientations of each read pair. All pairs with identical coordinates and orientations are analyzed and those having the highest sum of base qualities are classified as duplicates. It also removes duplicates from single-end libraries. Similarly, the *rmdup* function of *samtools* analyzes alignments obtained with a third-party tool to remove duplicates from both single- and paired-end reads. However, differently from Picard MarkDuplicates, *rmdup* is not able to remove interchromosomal duplicate reads. SEAL provides a distributed version of BWA [9] to perform the alignment and removes duplicates according to the same criteria employed by Picard MarkDuplicates.

Alignment-free tools detect duplicates by comparing read sequences. In particular, those reads characterized by a similarity score higher than a given threshold are classified as duplicates. Notably, tools that comply with this strategy are not affected by the bias introduced by a short-read mapping tool and can also be used in absence of a complete reference genome. Unfortunately, they may be computationally onerous, as each sequence of the dataset must be compared to all other sequences in the dataset. This is the reason why heuristics are defined and adopted to deal with the computational challenge.

Fastx-Toolkit Collapser [10], FastUniq [11], Fulcrum [12] and CD-HIT [13–15] are all examples of tools that implement an alignment-free strategy. Fastx-Toolkit Collapser is able to identify and remove identical sequences from single-end reads. Conversely, FastUniq has been designed to remove identical duplicates from paired-end reads. Removal is performed executing three steps in pipeline. Initially, all paired reads are loaded into memory. Then, read pairs are sorted according to their nucleotide sequences. Finally, duplicates are identified by comparing the adjacent read pairs in the sorted list. Fulcrum is able to identify identical and nearly-identical duplicates from both single- and paired-end reads. It identifies as potential duplicates those reads with an identical prefix of the nucleotide sequences. Potential duplicate reads are binned in different files, whose maximum size is user-defined. Read sequences within each file are compared to identify duplicates. CD-HIT provides two different tools to remove duplicates from single- and paired-end reads generated with 454 or Illumina platform. CD-HIT-454 analyzes libraries generated with 454 to identify duplicates that are either exactly identical or meet the following criteria: *a*) reads must be aligned at 5’-ends; *b*) for sequences of different length, a shorter read must be fully aligned with the longer one (the seed) and they have less than user-defined percentage of indels and substitutions. CD-HIT-DUP removes duplicates from Illumina libraries analyzing the prefix of the read sequences. Read sequences with identical prefix are considered duplicated. For paired-end reads, prefixes at both ends are checked. Features of the listed tools are summarized in Table 1.

**Table 1:** Summary of existing removal tools listed together with some of their relevant features

Tool	Strategy	Libraries	Platform	Type
Picard MarkDuplicates	alignment-based	single- and paired-end	Illumina and 454	nearly-identical
samtools rmdup	alignment-based	single- and paired-end	Illumina and 454	nearly-identical
SEAL	alignment-based	paired-end	Illumina	nearly-identical
FastX-Toolkit Collapser	alignment-free	single-end	Illumina and 454	identical
FastUniq	alignment-free	paired-end	Illumina and 454	identical
Fulcrum	alignment-free	single- and paired-end	Illumina and 454	nearly-identical
CD-HIT-454	alignment-free	single- and paired-end	454	nearly-identical
CD-HIT-DUP	alignment-free	single- and paired-end	Illumina	nearly-identical

The second column indicates the implemented strategy. The third column reports whether the corresponding tool support single- and/or paired-end read libraries. The fourth column reports the sequencing platforms supported. The fifth column reports whether the corresponding tool is able to remove only identical or nearly-identical duplicates

Recently, we proposed a new alignment-free method aimed at removing duplicate reads using Graphics Processing Units (GPUs) [16] generated with an Illumina platform. In particular, we implemented a prefix-suffix comparison algorithm which takes into account the per-base error rates generate with Illumina. The method consists of two phases, which have been massively parallelized on GPU. Initially, potential duplicate sequences are clustered according to their prefix. Then, the suffixes of the sequences in each cluster are compared to detect and remove duplicates.

Although the method can be efficiently used to remove both identical and nearly-identical duplicates, there are some constraints and limitations that need to be overcome. In particular, it does not allow to detect potential duplicates on prefixes longer than 27 bases, does not support paired-end read libraries, and imposes a constraint on the maximum size of the clusters.

In this work we present GPU-DupRemoval (standing for GPU-Duplicates Removal) a new implementation of our method devised to overcome these limitations. In particular, *i*) cluster reads without constraints on the maximum length of the prefixes are now allowed, *ii*) support for both single- and paired-end read libraries is provided, and *iii*) larger clusters of potential duplicates (without using heuristics) can now be processed.

## Implementation

Before going into relevant details of the proposed algorithm, let us give a short introduction to GPUs.

### Graphics processing units

GPUs are hardware accelerators that are increasingly used to deal with different computationally intensive bioinformatics algorithms (e.g., [17–21]). From an architectural perspective, the main difference between traditional CPUs and GPUs is related to the number of available cores. Indeed, the former are devices composed of few cores, with lots of cache memory able to handle a few software threads at a time. Conversely, the latter are devices equipped with hundreds of cores able to handle thousands of threads simultaneously, so that a very high level of parallelism can be reached.

The intensive use of GPUs over the last years has yielded a significant increases in the performance of several applications. However, it should be noted that only algorithms based on the SIMD paradigm can be effectively parallelized on GPUs. CPUs and GPUs should be considered as complementary for different types of processing. CPUs are optimized for flow control and low memory latency, whereas GPUs are optimized for data parallel computations. In this context, the GPU computing model uses CPUs and GPUs in a heterogeneous co-processing computing model. Computationally-intensive parts of an algorithm based on the SIMD paradigm can be accelerated by GPUs, whereas CPU is used to control the GPU execution while processing other parts of the algorithms not suitable for the GPU.

As for GPU programming, CUDA (Compute Unified Device Architecture) [22] and OpenCL (Open Computing Language) [23] offer two different interfaces for GPU programming. It is worth pointing out that OpenCL is an open standard that can be used to program CPUs, GPUs and other devices from different vendors whereas CUDA is specific to NVIDIA GPUs.

### The algorithm

Analysis of short-read datasets generated with Illumina highlighted a very low rate of indel errors (<0.01 *%*) while the number of occurrences of wrong bases increases with the base position [24]. Therefore, it is possible to deduce that: (*i*) the majority of duplicates will differ on few base substitutions; (*ii*) most of identical and nearly-identical duplicates are in fact characterized by an identical prefix. Starting from these considerations, we devised a method aimed at comparing only potential duplicate reads (i.e., reads with identical prefix) without taking into account indels in sequence comparisons.

**Fig. 1:**
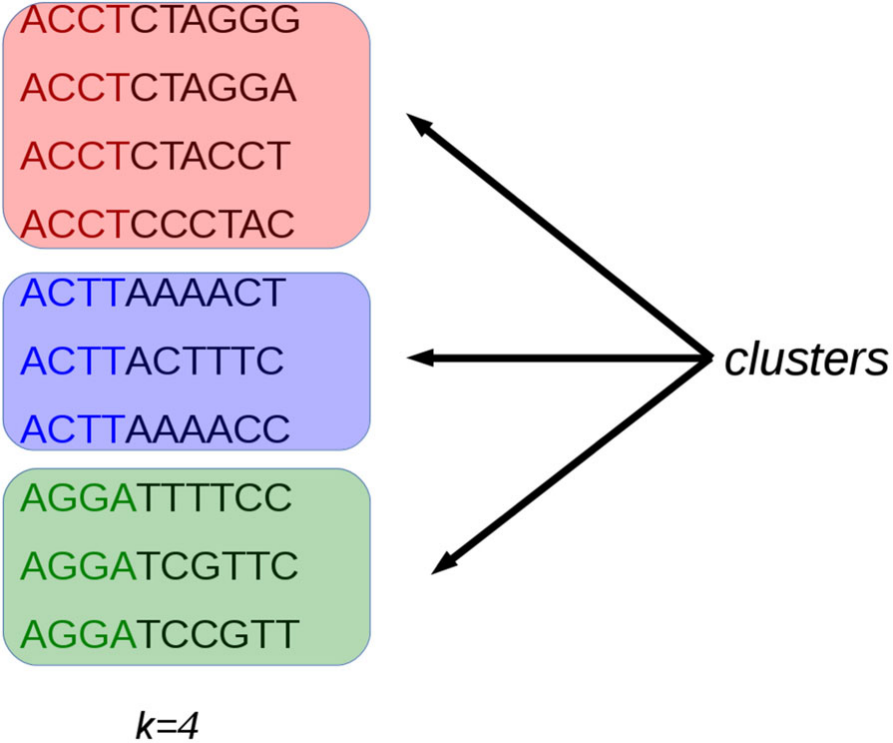
Clustering. Reads with an identical prefix of *k* nucleotides are considered potential duplicate reads. Image from [16] used under the terms of the Creative Commons Attribution License (CC BY)

**Fig. 2:**
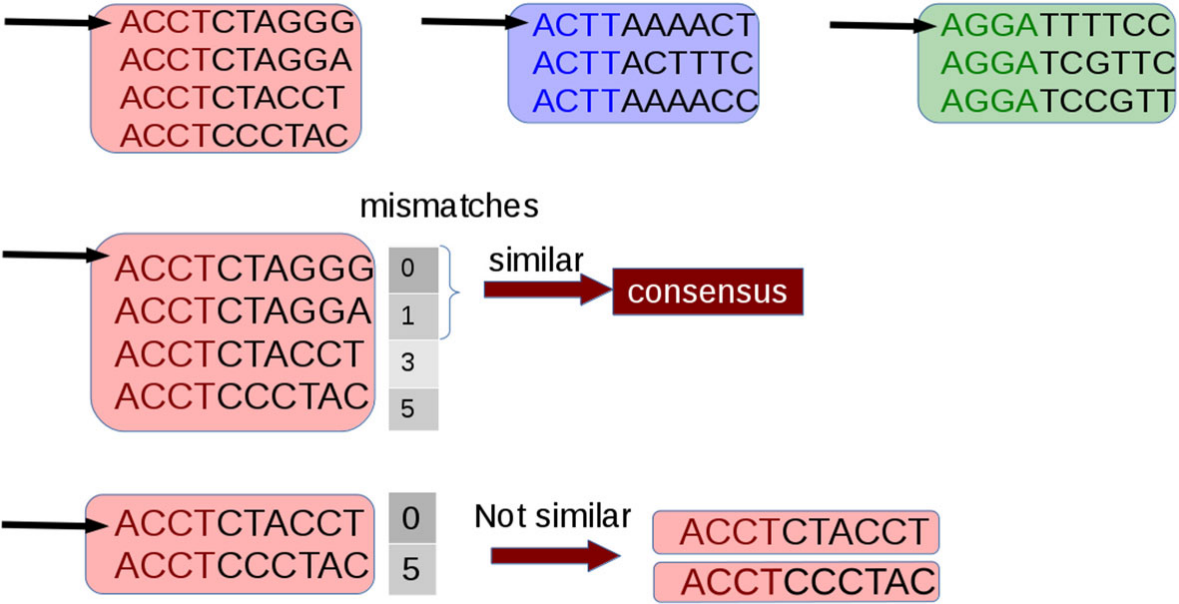
Comparison. The first read of each cluster is taken as a seed and its suffix is compared with that of the other sequences in the cluster. Sequences that differ from the seed for a number of mismatches lower than a user-defined threshold are considered duplicates of the seed. Each set of duplicates is removed from the cluster and are represented with a consensus sequence. The process is iterated until the cluster is empty. Image from [16] used under the terms of the Creative Commons Attribution License (CC BY)

Initially, potentially duplicated sequences are clustered together (see Fig. 1). Then, for each cluster, the first sequence is taken as a seed and its suffix is compared with those of the other sequences that fall in the selected cluster. Sequences that are identical or very similar to the seed are classified as duplicates. Duplicates are condensed in a new sequence and are removed from the cluster (see Fig. 2). Then, the process is iterated for the remaining sequences in the cluster (if any), until the cluster is empty or contains only one read sequence.

**Fig. 3:**
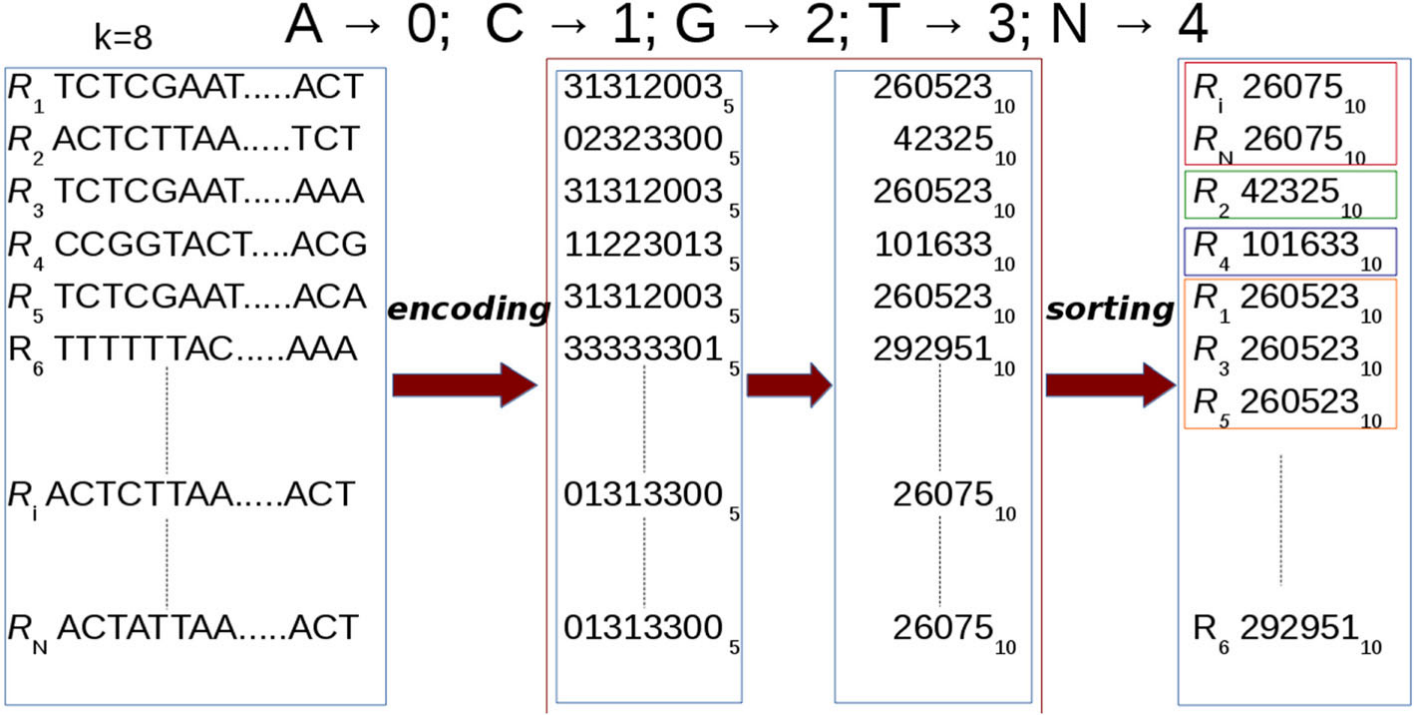
Enconding. Prefixes are subjected to a dual encoding. Initially, the nucleotides in a prefix are encoded with a numerical value from 0 to 4 (i.e., *A*→0, *C*→1, *G*→2, *T*→3, *N*→4). Then, these numerical representations are encoded using base-10. Finally, sorting is performed for clustering. In the figure, prefixes of length *k=8* are represented. Image from [16] used under the terms of the Creative Commons Attribution License (CC BY)

Clustering is performed by sorting the prefixes of the read sequences with our GPU-based CUDA-Quicksort [25]. As CUDA-Quicksort sorts numerical values, it is necessary to encode the prefixes of the read sequences. To this end, we devised the encoding with the aim to represent as many nucleotides as possible with a numeric value. In as doing, read sequence prefixes are subject to a dual numeric encoding. Initially, prefixes are represented with a base-5 encoding by replacing each nucleotide with a numerical value ranging from 0 to 4 (i.e., *A*→0,*C*→1,*G*→2,*T*→3,*N*→4). Representing items with *64 bit unsigned long long int* data type allows to encode and sort prefixes of up to 19 nucleotides. A longer prefix would exceed the limit for this type of data. It is possible to overcome this constraint using a different numerical base for representing prefixes. In particular, converting to base-10 the prefixes encoded using a base-5 encoding, it is possible to represent a number consisting of up 27 digits with a *64 bit unsigned long long int* (see Fig. 3).

For the sake of completeness, it should be pointed out that regarding the problem addressed in this work, quick-sort is more effective than other sorting algorithms, including radix-sort. With *k* number of digits in a key and *n* number of keys, the computational complexity of comparison-based radix-sort is *O*(*k*·*n*), whereas the complexity of quick-sort is *O*(*n*·*l* *o* *g*(*n*)). Hence, quick-sort outperforms radix-sort in the event that *k*>*l* *o* *g*(*n*), and viceversa. The performance of CUDA-Quicksort has been compared with Thrust Radix Sort [26], a cutting-edge algorithm running on GPUs. The comparative assessment has been made in the task of sorting items with long keys -characterized by 19 digits (i.e., the maximum number of digits used to represent the encoded read prefixes). Experiments, performed ensuring a uniform distribution on benchmark datasets (with varying size from 1M to 32M elements), show that CUDA-Quicksort outperforms Thrust Radix Sort with a speed-up ranging from 1.58x to 2.18x, depending on the dataset at hand [25].

Despite the fact that longer sequences (i.e., 27 instead of 19 nucleotides) can be processed, the latter encoding is still restrictive. As the quality of Illumina reads decreases with the position that a base has in the sequence being processed, more likely sequencing errors are localized towards the 3’ end of a read rather than in proximity of to the 5’ end. In GPU-DupRemoval, two sequences are classified as nearly identical if they fulfill a given constraint on the maximum number of allowed mismatches. In doing so, a mismatch is always considered a sequencing error, irrespective of the position (and of the quality) that a base has in a read. This processing policy gives rise to a fraction of natural nearly-identical sequences that may be erroneously classified as artificial duplicates. An approach to reduce the number of false positives in the process of duplicate identification is to limit the analysis of mismatches where is more likely sequencing errors are localized, choosing the prefix length according to the resulting quality scores obtained across all bases on the dataset. This length must be chosen to permit the selection of all bases whose average quality score is higher than a given threshold.

**Fig. 4:**
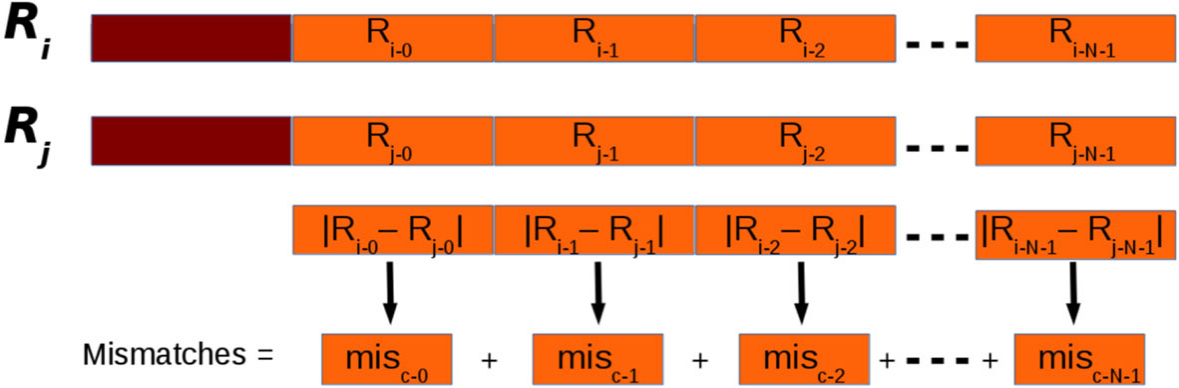
Suffixes. Suffixes (in *orange* in the figure) are analyzed in chunks. Each chunk is subject to the dual encoding used for prefixes (in *red* in the figure). The overall number of mismatches if obtained summing the partial number of mismatches obtained for each chunk. Image from [16] used under the terms of the Creative Commons Attribution License (CC BY)

After that reads have been clustered, their suffixes are compared. Basically, a base-per-base comparison of the nucleotides of the suffix of the seed with those of the other reads in a cluster should be performed in this phase. This approach might require a very high number of comparisons. Let *N* be the length of the suffixes, and let *m* be the minimum number of mismatches allowed to consider two sequences as not duplicated. In the best case, two sequences can be classified as not duplicated after *m* comparisons. In the worst case *N* comparisons must be performed. We implemented a different strategy aimed at reducing the number of comparisons. Initially, suffixes are split into fixed-length chunks. Each subsequence representative of a chunk is subjected to the same dual numeric encoding used to represent the prefixes for clustering. Then, for each cluster, the numerical difference between the *i-th* chunk of the seed and the related chunk of the other suffixes in a cluster is calculated (see Fig. 4). The order of magnitude of this difference provides information about the position of the leftmost different nucleotides. Then, subsequences are cut at the mismatch position. The rightmost parts of the mismatch position are maintained and the process is re-iterated. In the worst case, this approach is able to classify two sequences as not duplicated after *m* comparisons.

Suffix comparison has been massively parallelized on GPU. In particular, the chunks representatives of the reads in a cluster are loaded into the GPU memory and compared in parallel with the chunk of the seed. It should be noted that also the size of the clusters affects the overall computing time. In fact, depending on both the size of a cluster and the percentage of duplicates in it, a very high number of comparisons among sequences could be performed. In our method, very large clusters are split into smaller ones of fixed size with the aim of reducing the number of comparisons. In a similar manner, in Fulcrum, potential duplicates are binned in file of user-defined maximum size. On one hand this heuristic guarantees a reasonable computing time. However, on the other hand, it may affect the accuracy of the analysis. Therefore, in our view, resorting to this heuristic appears not appropriate. In the following, we describe the changes implemented in GPU-DupRemoval to cope with the constraints on the prefix length and the maximum size of a cluster, and to support paired-end reads.

#### Prefix length

The proposed clustering strategy resulted be very effective. Its computing time depends on the size of the dataset, whereas it does not depend on the prefixes length. Moreover, CUDA-Quicksort is able to cluster datasets of millions of reads very quickly. It should be pointed out that CUDA-Quicksort resulted be the faster implementation of the sorting algorithm on GPUs. In particular, it was up to 3 times faster than the CDP-Quicksort released by NVIDIA.

**Fig. 5:**
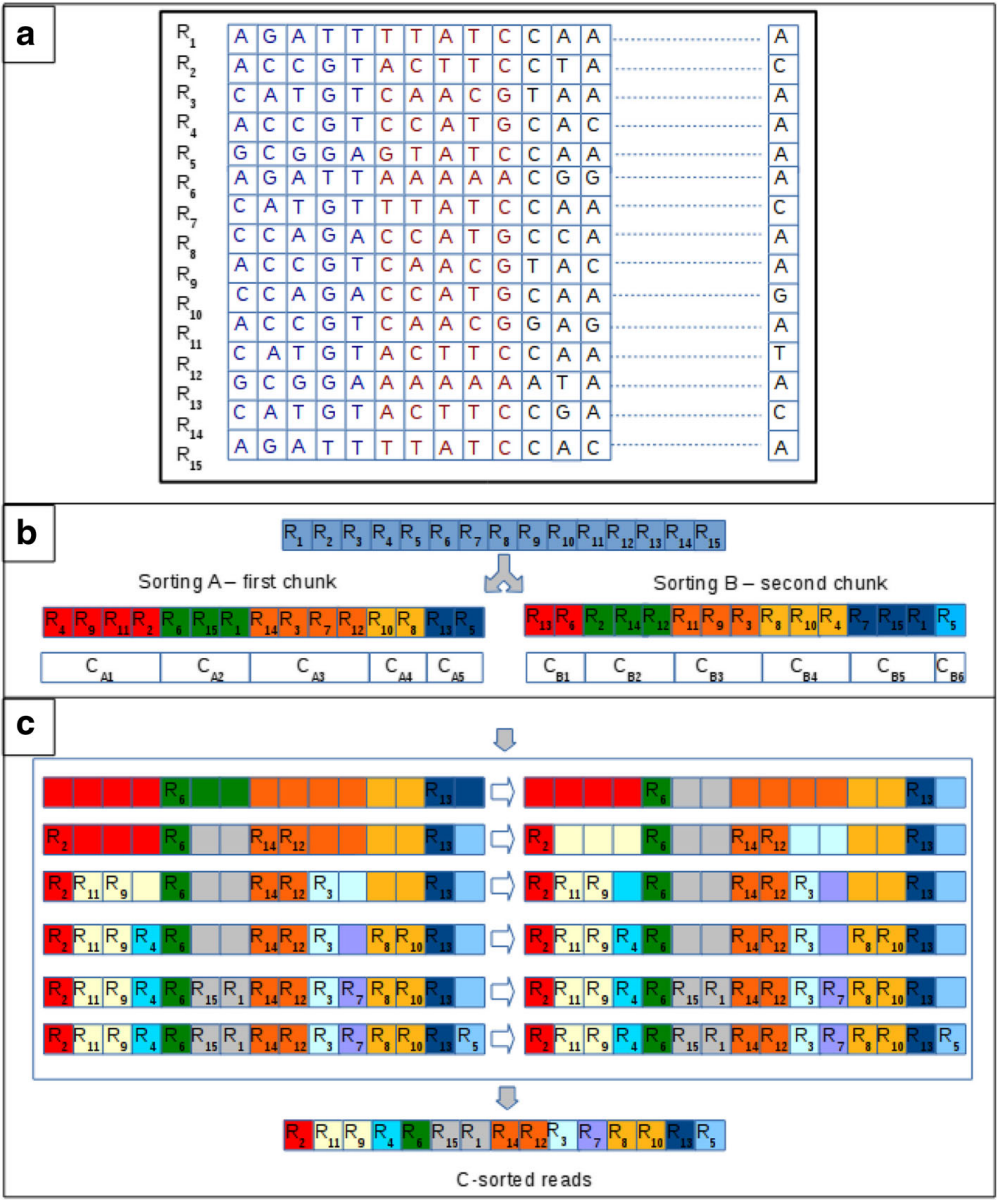
Multi-step clustering. To simplify the graphical representation, we assume that the multi-step clustering is enabled for prefixes longer than 5 nucleotides. In this example 15 reads are clustered analyzing prefixes of 10 nucleotides. **a** Initially, the prefixes are split into two chunks of 5 nucleotides. In the figure, the nucleotides of the first chunk are represented in blue, and those of the second chunk are represented in red. The clustering consists of three steps. **b** Reads are clustered by sorting them according to the first chunk of the prefixes (sorting *A* in the figure). Clustering generates 5 clusters of different size (*C*_*A*1_,*C*_*A*2_,*C*_*A*3_,*C*_*A*4_,*C*_*A*5_). Reads clustered together are represented with the same background color. Subsequently, reads are clustered by sorting them according to the second chunk of the prefixes (sorting *B* in the figure). This clustering generates 6 clusters unrelated from those of the previous clustering (*C*_*B*1_,*C*_*B*2_,*C*_*B*3_,*C*_*B*4_,*C*_*B*5_,*C*_*B*6_). **c** A new array is initialized and partitioned according to the size of the clusters of *A*. The sequences of each cluster in *B* are copied in the new array in the partition associated to their belonging cluster in *A*. Each read is copied into the first free position of the partition. The process is represented in the C box. Each row reported therein represents the process of copying the reads of a cluster in C. On the left it is shown where the reads are copied, whereas on the right it is shown how clusters are split after each iteration. Initially, the reads *R*_13_ and *R*_6_ of *C*_*B*1_ are copied in the new array. *R*_13_ belongs to *C*_*A*5_ in *A* and *R*_6_ belongs to *C*_*A*2_ in *A*. Being the first reads to be analyzed, they are copied in the first position related to its cluster in *A*. Cluster *C*_*A*2_ and *C*_*A*5_ are partially filled after this step. This implies that the reads in these clusters are not identical, according to the second chunk of their prefix. In fact, *R*_15_ and *R*_1_ have not been clustered together with *R*_6_ in *B*. Similarly, *R*_6_ has not been clustered together with *R*_13_ in *B*. Therefore, the clusters are split (see first row in C box). Cluster *C*_*A*2_ is split into two clusters. A cluster contains *R*_6_ and the other cluster (of size 2) is empty. Similarly, *C*_*A*5_ is split into two clusters of size 1. The process is iterated (as represented in the C box) until all clusters in *B* have been analyzed. The final sorting generates 11 clusters

Starting from these considerations, we devised a multi-step clustering strategy based on the existing one. For prefix length of up to 27 nucleotides clustering does not differ from the previous version of the tool. The approach differs when longer prefixes must be analyzed. Initially, prefixes are split into chunks of 27 nucleotides. Obviously, depending on the length of the prefixes, the last chunk might be shorter than 27 nucleotides. Each prefix is subjected to the dual numerical encoding previously described. Then, CUDA-Quicksort is used to sort reads according to two different criteria. The first sorting (say *A*) is obtained according to the first chunk of prefixes. It represents the partial sorting of the reads that will be iteratively updated to build the final sorting. The second sorting (say *B*) is obtained according to the second chunk of the prefixes. This sorting is used to update the first one in such a way that it becomes a sorting representative of both chunks. Basically, *B* is used to update the ordering of the reads in the clusters generated according to *A*. To this end, an array is initialized to store the new sorting (say *C*). The array will be partitioned taking into account the clusters generated with *A*. Then, according to *B*, the reads of each cluster are copied in the new array. Each read is copied in the first free position of the partition related to its belonging cluster generated with *A* (see Fig. 5). After that all reads in a cluster generated with *B* have been copied into the new array, both the number of clusters and their size is updated. A cluster is split into two clusters each time that its related partition in the new array is only partially written. At the end of the process, *A* is replaced by *C*. Then, the process is re-iterated with the following chunks (if any).

It might seem that the described algorithm implements a radix sort. However, there are considerably differences between the two algorithms. Radix-sort is a non-comparative sorting algorithm that performs a digit-by-digit sorting on keys. In the proposed multi-step clustering strategy, GPU-DupRemoval uses CUDA-Quicksort to perform (at each step) a comparison-based sorting of numbers (not digits), representative of sub-sequences of the available reads. Furthermore, let us recall that two variants of the radix-sort (RS) exist, able to process keys from the less to the most significant digit (i.e., LSD/RS) and from the most to the less significant digit (i.e., MSD/RS). The apparent resemblance of multi-step clustering algorithm with radix-sort in fact applies more to LSD/RS than to MSD/RS. However, only MSD/RS could be used to address the problem at hand as, unlike LSD/RS, MSD/RS dispatches all digits with identical value into a specific bucket and recursively repeats the same operation with all buckets, until sorting is complete. Notably, keys that occur in a bucket are sorted independently from those in other buckets. Conversely, in the proposed approach, sorting is performed by analyzing all sequences at each step.

This multi-step clustering strategy has been used to optimize also the removal of identical duplicate reads. In the first implementation the same approach was used to remove identical and nearly-identical reads. However, identical reads can be removed more easily than nearly-identical ones. In particular, identical reads can be identified by clustering the reads by their entire sequences. In GPU-DupRemoval identical duplicates are automatically removed by clustering reads according to their entire sequences. It should be pointed-out that the multi-step clustering is not used to remove identical duplicates when GPU-DupRemoval is run to remove both identical and nearly-identical read sequences. It is solely used when GPU-DupRemoval is used to remove identical reads.

#### Maximum size of a cluster

As previously described, in the first implementation of our method large clusters are automatically split into smaller. On one hand, analyzing small clusters may improve the performance in terms of computing time; on the other hand, it may worsen the performance in terms of accuracy. The smaller the cluster is, the faster the processing is, as fewer comparisons are required. Unfortunately, duplicates separated during the splitting will be not identified.

The problem has been addressed in GPU-DupRemoval, which is able to analyze large clusters without the need for splitting them. Originally, only a level of parallelism was implemented. At each iteration, the first read in each cluster (i.e., the seed) is compared with the other reads in the cluster. Depending on both the size of a cluster and on the percentage of duplicates, this approach may require many iterations. Let *N* be the size of the cluster, when each read is uniquely represented in the cluster (worst case), *N-1* iterations must be performed. This computational challenge can be efficiently addressed by adding a second level of parallelism aimed at comparing multiple seeds of a cluster in a single iteration. In the current implementation, at each iteration the possibility to compare in parallel multiple seeds of a cluster is assessed. It should be pointed out that depending on the size of the dataset at hand, an iteration might require one or more kernel launches. Notably, GPU-DupRemoval applies different strategies, depending on the number of kernel launches required to analyze the dataset. Initially, GPU-DupRemoval determines the thread block size, the number of thread blocks and the GPU memory required to analyze the given dataset and to compare the read chunks that occur in each cluster with a single seed. As long as more than one kernel launch is required in an iteration, GPU-DupRemoval compares reads that occur in a cluster with a single seed. Conversely, when a single kernel launch is made in an iteration, GPU-DupRemoval checks the feasibility of comparing multiple seeds in parallel. The upper limit of seeds that can be compared in parallel for each cluster (say *n*) is determined according to the constraints on the maximum number of blocks that can be created per kernel launch and on the memory of the device. At each iteration, up to *n* seeds are compared in parallel for each cluster. The upper limit for the seeds that can be compared in parallel will increase with a decrease of the read sequences to be analyzed. As the number of reads decreases at each iteration, the value of *n* is re-calculated after each kernel launch. In so doing, when multiple seeds are analyzed in parallel, a read is compared with two or more seeds in the same iteration, and, depending on the results of comparisons, it may be classified as duplicated of two or more seeds. In these cases, the read will be considered as duplicated of the first seed.

#### Supporting paired-end reads

**Fig. 6:**
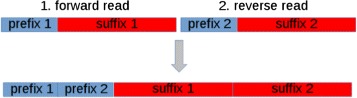
Merging paired-end reads. Paired-end reads with identical prefix at both ends can be considered potential duplicates. The same clustering strategy used to identify potential duplicates in single-end reads can also be used for paired-end reads. In this case, paired-end reads need to be merged as represented in the figure. A sequence representative of a pair is obtained by merging the prefixes and the suffixes of both forward and reverse read. With *N* the length of the read sequence and *p* length of the prefixes, the new sequence consists of 2·*N* nucleotides and is represented by a prefix of 2·*p* nucleotides

**Fig. 7:**
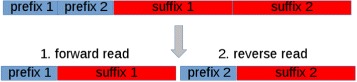
De-merging paired-end reads. After that duplicates have been removed, sequences are demerged to generate both forward and reverse reads

GPU-DupRemoval has been devised to support both single- and paired-end reads. It should be noted that duplicates from paired-end reads can be removed similarly to that concerning single-end reads. In fact, paired-end reads with an identical prefix at both ends can be considered as potential duplicates. Hence, potential duplicates can be identified by clustering reads according to the prefixes that occur at both ends. To this end, GPU-DupRemoval builds a new sequence representative of both reads for each pair in the dataset. Each sequence is build by merging separately prefixes and suffixes, as shown in Fig. 6. Subsequently, these sequences are analyzed according to the same method used for single-end reads. Finally, after that duplicates have been removed, sequences are demerged (see Fig. 7). It should be pointed out, that this strategy to support paired-end reads is well suited to the current implementation of the algorithm that gives a viable solution to the issue concerning the maximum length of prefixes. The limitation on the length of the prefixes of the previous implementation would negatively affect the capability of removing duplicates from paired-end reads.

## Results and discussion

Experiments have been designed to assess the performances of GPU-DupRemoval to remove identical and nearly-identical duplicates from single- and paired-end read libraries, with both synthetic and real life data. In this section, we first introduce experiments on synthetic data, which are mainly aimed at assessing the reliability of GPU-DupRemoval. Experimental results obtained on real data are reported afterwards.

*Performance evaluation on synthetic data*

Synthetic libraries have been generated with the Sherman simulator [27]. It should be pointed out in advance that Sherman does not permit to set the percentage of duplicates in a library. Initially, we used Sherman to generate a library consisting of 750 thousands of 100 bp single-end reads. Subsequently, synthetic reads have been processed to generate 250 thousands of 100 bp duplicate reads. Duplicates consist of identical duplicates (100 thousands), duplicates generated by simulating a 1 % of sequencing error (100 thousands), and duplicates generated by simulating a sequencing error ranging between 2 and 3 % (50 thousands). Similarly, we generated a library consisting of 1 millions of 100 bp paired-end reads. In this case, the sequencing error has been uniformly simulated on both ends. As for both single- and paired-end reads, duplicates have been generated by simulating sequencing errors using an error rate curve that follows an exponential decay model, with the aim of mimicking real data.

As for the single-end library, GPU-DupRemoval has been compared with Fastx-Toolkit Collapser, CD-HIT-DUP, and Fulcrum. Experiments have been defined to assess the reliability of the tool to identify and remove duplicates according to the sequencing errors injected therein. Results reported in Table 2 show the percentage of reads removed from each tool when used to identify duplicates according to a sequencing error ranging from 0 to 3 %. Apart from CD-HIT-DUP, the other tools work properly and have been able to identify all duplicates. Similar behavior has been observed for paired-end reads (see Table 3). In this case, GPU-DupRemoval has been compared with FastUniq, CD-HIT-DUP, and Fulcrum. It should be pointed out that both Fastx Toolkit Collapser and FastUniq does not support removal of nearly-identical reads.

**Table 2:** Percentage of removed duplicates are reported, varying the allowed number of differences from 0 to 3 mismatches for a synthetic single-end read library consisting of 1 millions of 100 bp reads. The library consists of 25 % of duplicates

	Mismatches			
Tool	0	≤1	≤2	≤3
GPU-DupRemoval	10.0 %	20.0 %	22.5 %	25.0 %
CD-HIT-DUP	10.0 %	10.0 %	16.0 %	25.0 %
Fastx-Toolkit Collapser	10.0 %	-	-	-
Fulcrum	10.0 %	20.0 %	22.5 %	25.0 %

Clustering for GPU-DupRemoval and Fulcrum has been performed analyzing prefixes of 25 bases when used to remove nearly-identical duplicates. As for identical duplicates clustering has been performed on the entire length of the reads for both tools. It should be pointed out that GPU-DupRemoval automatically clusters the reads according to their length when used to remove identical duplicates. Tool settings: *i*) GPU-DupRemoval *-g 0 -D 0* (for identical duplicates) and *-g 0 -p 25 -D <nb_of_mismatches >* (for nearly-identical duplicates); *ii*) CD-HIT-DUP *-u 0 -c <nb_of_mismatches* >; *iii*) Fulcrum *-b <prefix_length > -s -t s* (for clustering) and -q 0 -s -t s -c <nb_mismatches >. <nb_of_mismatches >: the allowed number of mismatches. It has been set to 0, 1, 2, 3 for the different experiments. <prefix_length > was set to 100 for identical duplicates and to 25 for nearly-identical duplicates. Fastx-Toolkit Collapser does not require any parameter apart those aimed at specifying the input and the output files

**Table 3:** Table reports the percentage of removed duplicates varying the allowed number of difference from 0 to 3 mismatches for a synthetic paired-end read library consisting of 1 millions of 100 bp reads. The library consists of 25 % of duplicates

	Mismatches			
Tool	0	≤1	≤2	≤3
GPU-DupRemoval	10.0 %	20.0 %	22.5 %	25.0 %
CD-HIT-DUP	10.0 %	10.0 %	16.2 %	17.5 %
FastUniq	10.0 %	-	-	-
Fulcrum	10.0 %	20.0 %	22.5 %	25.0 %

Tool settings: *i*) GPU-DupRemoval *-g 0 -D 0* (for identical duplicates) and *-g 0 -p 10 -D <nb_of_mismatches >* (for nearly-identical duplicates); *ii*) CD-HIT-DUP *-u 0 -c <nb_of_mismatches >*; *iii*) Fulcrum *-b <prefix_length > -s -t p* (for clustering) and -q 0 -s -t p -c <nb_mismatches >. As for Fulcrum, <prefix_length > was set to 100 for identical duplicates and to 25 for nearly-identical duplicates. FastUniq does not require specific parameters apart from those aimed at specifying input and output files

*Performance evaluation on real data*

To assess the performance of GPU-DupRemoval on real data, we used it to remove duplicates of two libraries generated with the Illumina platform; i.e., library *SRR921897* consisting of 50 millions of 100 bp single-end reads, and library *SRR005718* consisting of 32 millions of 36 bp paired-end reads. Experiments have been carried-out to identify and remove identical and nearly-identical duplicates with up to 1 and 3 mismatches.

Experiments described hereinafter have been carried out on a 12 cores Intel Xeon CPU E5-2667 2.90 GHz with 128 GB of RAM. An NVIDIA (Kepler architecture based) Tesla k20c card with 0.71 GHz clock rate and equipped with 4.8 GB of global memory has been used to execute GPU-DupRemoval.

Experiments have been designed with the goal of providing a rigorous comparison among the tools. In this context, it should be pointed out that Fulcrum considers as a*N*y those bases with a quality score under a user defined-threshold. Being not supported by the other tools, this option has been disabled as it can affect the percentage of duplicates removed. Moreover, differently from the other tools, Fulcrum parallelizes the computation on multiple CPU cores. Therefore, to provide a rigorous comparison in terms of computing time with GPU-DupRemoval, Fulcrum has been run parallelized on all available CPU cores.

Identical parameters have been used to perform the clustering in both GPU-DupRemoval and Fulcrum. Clustering has been performed according to different lengths of the prefixes with the aim to show how this parameter affects the removal of duplicates. As for nearly-identical duplicates, clustering has been performed by analyzing prefixes of 25/35/45/55 bases for *SRR921897* and prefixes of 10/15 bases for *SRR005718*. As for identical duplicates, GPU-DupRemoval automatically clusters the reads by their entire nucleotide sequences. The same constraint has been used in Fulcrum to remove identical duplicates.

Table 4 summarize the results obtained by removing duplicates from the *SRR921897* library in terms of removed reads, computing time, and peak of memory required. GPU-DupRemoval, CD-HIT-DUP, and Fastx Toolkit Collapser removed the same percentage of identical read sequences (7.4 %). A slightly lower amount (7.3 %) of identical reads has been removed by Fulcrum. As for nearly-identical reads, Fulcrum removed a slightly higher amount of sequences with respect to GPU-DupRemoval. In particular, clustering the reads with a prefix length of 25/35/45/55 bases GPU-DupRemoval removed 9.2/8.9/8.7/8.4 % of nearly-identical sequences with up to 1 mismatch, whereas the percentage reached by Fulcrum was 9.8/9.6/9.4/9.2 %. Similar results have been obtained to remove nearly-identical sequences with up to 3 mismatches. In this case, clustering the reads with a prefix length of 25/35/45/55 bases GPU-DupRemoval removed 12.2/11.5/10.8/10.0 % of sequences, whereas the percentage of sequences removed by Fulcrum was 13.1/12.3/11.7/10.9 %. As for CD-HIT-DUP, it has been able to remove 8.0/9.8 % of duplicates with up to 1/3 mismatches.

**Table 4:** Performance comparison on the *SRR921897* library among GPU-DupRemoval, Fastx Toolkit Collapser, CD-HIT-DUP, and Fulcrum

Tool	Prefix length	Mismatches	Removed	Time	Memory
GPU-DupRemoval ^1^	100	0	7.4 %	4 m	13.1 GB
	25	1	9.2 %	18 m	16.6 GB
		3	12.2 %	17 m	16.6 GB
	35	1	8.9 %	12 m	17.2 GB
		3	11.5 %	11 m	17.2 GB
	45	1	8.7 %	7 m	16.5 GB
		3	10.8 %	7 m	16.5 GB
	55	1	8.4 %	6 m	17.5 GB
		3	10.0 %	5 m	17.5 GB
GPU-DupRemoval ^2^	25	0	7.4 %	22 m	16.6 GB
		1	9.0 %	18 m	16.6 GB
		3	12.0 %	15 m	16.6 GB
CD-HIT-DUP	N/A	0	7.4 %	17 m	33.3 GB
		1	8.0 %	15 m	49.2 GB
		3	9.8 %	28 m	53.5 GB
Fulcrum	100	0	7.3 %	53 m	1.8 GB
	25	1	9.8 %	47 m	1.8 GB
		3	13.1 %	57 m	1.8 GB
	35	1	9.6 %	36 m	2.3 GB
		3	12.3 %	37 m	1.6 GB
	45	1	9.4 %	28 m	1.9 GB
		3	11.7 %	29 m	1.7 GB
	55	1	9.2 %	25 m	2.1 GB
		3	10.9 %	26 m	2.3 GB
Fastx Toolkit Collapser	N/A	0	7.4 %	12 m	10.2 GB

As for GPU-DupRemoval the table reports the results for both the current (GPU-DupRemoval ^1^) and the first implementation (GPU-DupRemoval ^2^) of the algorithm. The library consists of 49.999.923 of 100 bp single-end reads generated with Illumina platform. The first column reports the name of the tool. The second column reports the prefix length used for clustering the reads for GPU-DupRemoval and Fulcrum. The third column reports the constraint on the allowed number of mismatches. The fourth column reports the percentage of reads that have been removed. The fifth and sixth column report the computing time and the peak of memory required to perform the experiment. Tool settings: *i*) GPU-DupRemoval ^1^ *-g 0 -D 0* (for identical duplicates) and *-g 0 -p <prefix_length > -D <nb_mismatches >* (for nearly-identical duplicates); *ii*) GPU-DupRemoval ^2^ *-g 0 -p 25 -D <nb_mismatches >*; *iii*) CD-HIT-DUP *-u 0 -c <nb_of_mismatches >*; *iv*) Fulcrum *-b <prefix_length > -s -t s* (for clustering) and -q 0 -n 12 -s -t s -c <nb_mismatches >. <prefix_length > was set to 100 for identical duplicates and to 25/35/45/55 for nearly-identical duplicates. No parameter is required for Fastx Toolkit Collapser

We deem that this slight discrepancy on the percentage of sequences removed by GPU-DupRemoval and Fulcrum depends on the different strategies implemented to compare the reads in a cluster. In Fulcrum, initially, a list of groups of strongly similar reads is initialized using the first read of each cluster (say *r*). Subsequently, each read in a cluster is compared to *r* and if considered similar to *r* it is added to the group and a new consensus sequence *r* is calculated and used for the following comparison. Unlike, GPU-DupRemoval does not compare the reads in a cluster with a consensus sequences. In GPU-DupRemoval duplicates are detected by comparing each read in a cluster to all other sequences in the cluster.

Results show how the prefix length used for clustering can affect the percentage of removed reads. Analysis of results show that the percentage of sequences classified as duplicates decreases with increasing the prefix length. For instance, with a prefix of 35 bases the percentage of nearly-identical sequences with up to 1/3 mismatches removed by GPU-DupRemoval decreased of 0.3/0.7 % with respect to the percentage obtained using a prefix of 25 bases. Similar results have been obtained by Fulcrum.

Performance in terms of computing time shows that GPU-DupRemoval is the fastest tool. It resulted to be 3.0x faster than Fastx Toolkit Collapser in removing identical duplicates, whereas it resulted to be up to 4.2x/2.1x/5.6x faster than CD-HIT-DUP and up to 13.2x/4.1x/5.2x faster than Fulcrum to remove duplicates with up to 0/1/3 mismatches.

As for memory consumption, Fulcrum outperforms the other tools, the worst being CD-HIT-DUP, which requires a notable high amount of memory. Experiments show that the memory required increases with the allowed number of mismatches. In particular, CD-HIT-DUP required 33.3 GB of memory to remove identical duplicates and 49.2/53.5 GB of memory to remove duplicates with up to 1/3 mismatches. As for GPU-DupRemoval, the amount of memory required depends on both the size of the dataset and the type of duplicates removed, whereas it is unrelated from the number of differences allowed among duplicates. To perform the experiments, GPU-DupRemoval required 13.1 GB to remove identical duplicates and 16.6/17.2/16.5/17.5 GB to remove nearly-identical sequences clustering the reads with a prefix length of 25/35/45/55 bases.

Experiments have also been performed to compare the performance of the current implementation of GPU-DupRemoval with that obtained with the previous release. Experiments with the first release of the algorithm has been performed clustering the reads with a prefix length of 25 bases. As for identical duplicates, both releases of the tool have been able to remove the same percentage of identical duplicates. However, due to the multi-clustering strategy the current release of the tool resulted to be 5.5x faster than the previous implementation. As for nearly-identical reads both versions of the tool exhibit comparable performance in terms of computing time and memory consumption. However, the current version of the tool, which is able to analyze large clusters without heuristics, has been able to remove a slightly higher percentage of duplicates.

Similar results are reported in Table 5 for *SRR005718* library. As for identical duplicates, all tools removed the same amount of reads (2.9 %). GPU-DupRemoval and Fulcrum show almost identical results. GPU-DupRemoval removed 3.6/3.5 % of duplicates with up to 1 mismatch clustering the reads with a prefix length of 10/15 bp, whereas Fulcrum removed always 3.6 % of duplicates. As for nearly-identical duplicates with up to 3 mismatches GPU-DupRemoval removed 4.0/3.9 % of duplicates clustering the reads with a prefix length of 10/15 bases, whereas Fulcrum removed 4.2/4.1 % of duplicates. As for CD-HIT-DUP, it removed 3.3/3.0 % of duplicates with up to 1/3 mismatches.

**Table 5:** Performance comparison on the *SRR005718* library among GPU-DupRemoval, FastUniq, CD-HIT-DUP, and Fulcrum. The library consists of 32.160.546 of 36 bp paired-end reads generated with an Illumina platform

Tool	Prefix length	Mismatches	Removed	Time	Memory
GPU-DupRemoval	36	0	2.9 %	5 m	6.6 GB
	10	1	3.6 %	5 m	8.2 GB
		3	4.0 %	4 m	8.2 GB
	15	1	3.5 %	5 m	6.9 GB
		3	3.9 %	4 m	6.9 GB
CD-HIT-DUP	N/A	0	2.9 %	6 m	26.9 GB
		1	3.3 %	8 m	35.2 GB
		3	3.0 %	11 m	37.7 GB
Fulcrum	36	0	2.9 %	35 m	720 MB
	10	1	3.6 %	1h 4 m	720 MB
		3	4.2 %	1h 10 m	720 MB
	15	1	3.6 %	34 m	1.4 GB
		3	4.1 %	36 m	1.0GB
FastUniq	N/A	0	2.9 %	6 m	10.1 GB

The first column reports the name of the tool. The second column reports the prefix length used for clustering the reads for GPU-DupRemoval and Fulcrum. The third column reports the constraint on the allowed number of mismatches. The fourth column reports the percentage of reads that have been removed. The fifth and sixth column report the computing time and the peak of memory required to perform the experiment. Tool settings: *i*) GPU-DupRemoval *-g 0 -D 0* (for identical duplicates) and *-g 0 -p <prefix_length > -D <nb_of_mismatches >* (for nearly-identical duplicates); *ii*) CD-HIT-DUP *-u 0 -c <nb_of_mismatches >*; *iii*) Fulcrum *-b <prefix_length > -s -t p* (for clustering) and -q 0 -n 12 -s -t p -c <nb_mismatches >. <prefix_length > was set to 36 for identical duplicates and to 10/15 for nearly-identical duplicates

As for computing time, the performance of GPU-DupRemoval to remove identical reads are similar with those obtained by CD-HIT-DUP and FastUniq, whereas it resulted to be 7.0x faster than Fulcrum. As for nearly-identical duplicates GPU-DupRemoval resulted be up to 1.6x/2.3x faster than CD-HIT-DUP, and up to 12.8x/17.5x faster than Fulcrum to remove duplicates with up to 1/3 mismatches.

As for memory consumption, a behavior similar to the one observed on the *SRR921897* library has been observed. In particular, Fulcrum outperforms all remaining tools, requiring in the worst case 1.4 GB of memory, whereas CD-HIT-DUP required 26.9/35.2/37.7 GB of memory to remove duplicates with up to 0/1/3 mismatches. As for GPU-DupRemoval, it required 6.6 GB of memory to remove identical duplicates, and 8.2/6.9 GB to remove nearly-identical ones by clustering the reads with a prefix of 10/15 bases.

## Conclusions

In this work we presented GPU-DupRemoval, a tool aimed at removing identical and nearly-identical duplicates from sequencing libraries generated with Illumina platforms. GPU-DupRemoval implements an alignment-free strategy and exploits the computational power of modern GPUs to remove duplicates from both single- and paired-end libraries.

Experimental results show that GPU-DupRemoval is very effective at removing duplicate reads, as it outperforms almost all analyzed tools. In terms of ability to identify and remove duplicates, its performance are comparable with that of Fulcrum. However, it resulted to be very faster than Fulcrum, especially at removing duplicates from paired-end reads.

The current implementation of GPU-DupRemoval overcomes almost all limitations highlighted with respect to its first implementation. Currently, the constraint on maximum size of the sequencing library still holds. As highlighted in the previous work, sorting requires all prefixes to be loaded into the memory of the GPU device. Therefore, the maximum size of the library that can be analyzed depends on the memory of the GPU card used.

## Availability and requirements

**Project name:** GPU-DupRemoval **Project home page:** http://www.itb.cnr.it/web/bioinformatics/gpu-dupremoval **Operating system(s):** Linux**Programming language:** CUDA C**Other requirements:** NVIDIA GPU card with compute capability ≥ 3.5**License:** free for academic use**Any restrictions to use by non-academics:** license needed

## Abbreviations

CUDA: Compute unified device architecture
GPU: Graphics processing units
NGS: Next generation sequencing
